# xECGArch: a trustworthy deep learning architecture for interpretable ECG analysis considering short-term and long-term features

**DOI:** 10.1038/s41598-024-63656-x

**Published:** 2024-06-07

**Authors:** Marc Goettling, Alexander Hammer, Hagen Malberg, Martin Schmidt

**Affiliations:** Institute of Biomedical Engineering, TU Dresden, Fetscherstr. 29, 01307 Dresden, Germany

**Keywords:** Cardiology, Biomedical engineering, Computer science

## Abstract

Deep learning-based methods have demonstrated high classification performance in the detection of cardiovascular diseases from electrocardiograms (ECGs). However, their blackbox character and the associated lack of interpretability limit their clinical applicability. To overcome existing limitations, we present a novel deep learning architecture for interpretable ECG analysis (xECGArch). For the first time, short- and long-term features are analyzed by two independent convolutional neural networks (CNNs) and combined into an ensemble, which is extended by methods of explainable artificial intelligence (xAI) to whiten the blackbox. To demonstrate the trustworthiness of xECGArch, perturbation analysis was used to compare 13 different xAI methods. We parameterized xECGArch for atrial fibrillation (AF) detection using four public ECG databases ($n = 9854$ ECGs) and achieved an F1 score of 95.43% in AF versus non-AF classification on an unseen ECG test dataset. A systematic comparison of xAI methods showed that deep Taylor decomposition provided the most trustworthy explanations ($+24\%$ compared to the second-best approach). xECGArch can account for short- and long-term features corresponding to clinical features of morphology and rhythm, respectively. Further research will focus on the relationship between xECGArch features and clinical features, which may help in medical applications for diagnosis and therapy.

## Introduction

Deep learning (DL) algorithms show high classification performance in automatic disease detection from biosignals^1^. In particular, the detection of cardiovascular diseases (CVDs) based on the electrocardiogram (ECG) is of great interest as the global burden of CVD increases^2^.

Despite the competitive performance of DL algorithms, reaching classification performances in the range of general practitioners and exceeding these of nurses^3^, the integration into clinical routine is not very advanced. One reason is the blackbox character of DL approaches. Due to the highly non-linear behavior, the models’ reasoning is seemingly impossible to understand and thus unjustifiable for medical decision-making. However, concepts from the field of explainable artificial intelligence (xAI) might bridge the gap between current research and clinical applications. Most commonly for DL, xAI algorithms establish explainability in the field of time series analyses by highlighting the relevance of samples for the classification of the time series in a so-called saliency map or heatmap (e.g.^4–7^). Currently, multiple methods with different underlying concepts of explanation generation exist, like attention masks^8^, gradient-based sensitivity analysis^9^, decomposition-based attribution analysis^10^, and perturbation-based analysis^11^ but their current use in classification frameworks lacks interpretability and therefore trustworthiness for clinical applications.

Trustworthiness is given when relevant xAI identified features match previous clinical expertise^12^. However, ECG characteristics of clinical expertise can be divided into the short-term (morphological) and the long-term (rhythmic) domain^13^. Depending on the pathophysiology, either one or both of the two domains show specific variations. Therefore, characteristics of both domains can be handled separately or combined for decision-making. A trustworthy explainable DL architecture should therefore be able to represent characteristics of both domains to be useful in clinical decision-making.

Atrial fibrillation (AF) is the most common cardiac arrhythmic disease globally and leads to serious health consequences including premature death^14^. It is defined by characteristics of the short- and long-term domain as fibrillatory waves (F waves) lead not only to a morphological beat deformation but also to irregular excitation transmission to the ventricles (absolute arrhythmia)^14^. Because of the well-practiced clinical expertise and the real-world relevance, AF is best suitable for developing a novel DL architecture considering both short- and long-term characteristics.

**Table 1:** Overview of works focusing on AF detection by machine and deep learning algorithms. Accuracy (Acc.), sensitivity (Sens.), and specificity (Spec.) scores of works that reported scores for multiple datasets were averaged over all datasets. AF, atrial fibrillation; NSR, normal sinus rhythm; AFL, atrial flutter; J, junctional rhythm; DWT, discrete wavelet transformation; SVM, support vector machine; HRV, heart rate variability; CWT, continuous wavelet transformation; CNN, convolutional neural network; DDNN, deep densely connected neural network; H-ELM, hierarchical extreme learning machine; CS, compressed sensing; LSTM, long short-term memory; XGBoost, extreme gradient boosting.

Author	Year	ECG	Class. task	Approach	Database	Acc.	Sens.	Spec.
Asgari et al.^64^	2015	1-lead	AF, non-AF	DWT, SVM	MIT-BIH AF	97.1	97.0	97.1
Andersen et al.^65^	2017	1-lead	AF, non-AF	HRV, SVM	MIT-BIH AF	96.4	96.8	96.2
Wu et al.^66^	2019	1-lead	NSR, AF, OTHER, NOISE	CWT, CNN	MIT-BIH AD/MVAD/AF/NSR/NST	97.6	97.6	99.2
Cai et al.^38^	2020	12-lead	AF, non-AF	DDNN	Chinese PLA GH Wearable, CPSC 2018	98.2	96.5	98.7
Ghosh et al.^67^	2020	1-lead	AF, NSR	Cosine filter bank, H-ELM	MIT-BIH AF/AD	99.4	98.8	100
Nurmaini et al.^68^	2020	1-lead	AF, NSR	CNN	CinC Challenge 2017, MIT-BIH AF/MVAD	94.9	95.5	95.5
Zhang et al.^69^	2020	1-lead	AF, non-AF	CS, CNN	MIT-BIH AF	96.2	95.9	96.5
Jo et al.^15^	2021	1-lead	AF, non-AF	CNN feature modules	Sejong ECG, PTB-XL, Chapman, CinC Challenge 2017	98.9	99.0	98.9
Petmezas et al.^70^	2021	1-lead	AF, NSR, AFL, J	CNN, LSTM	MIT-BIH AF	97.4	97.0	98.4
Serhal et al.^71^	2023	1-lead	AF, NSR	EMD, CNN	PTB-XL	98.8	-	-
Choi et al.^72^	2024	1-lead	AF, non-AF	LSTM, XGBoost	PTB-XL, Chapman	93.00	95.35	89.56
Our approach	2024	1-lead	AF, non-AF	CNN ensemble	PTB-XL, Georgia, CPSC 2018, Chapman	95.33	94.87	95.82

Table 1 contains an overview of works with representative classification scorings. Multiple machine learning (ML) and DL approaches for AF detection are proposed with high classification performances, reaching sensitivities and specificities over 90%. As xAI approaches are new in the field of DL-based biosignal analyses, only few studies employing different xAI methods for ECG classification exist. Jo et al.^15^ built a convolutional neural network (CNN) architecture consisting of different submodules for the detection of features like P wave presence or RR irregularity and interpreted the results by feature-module specific gradient class activation maps (GradCAM). GradCAM was also used to explain architectures for detecting CVDs^4–6,16,17^. Honarvar et al.^18^ applied DeepLIFT for left ventricular dysfunction detection explanation. Strodthoff et al.^19^ showed examples of explanations by a layer-wise relevance propagation (LRP) rule for a CNN trained for ECG classification, while Salinas-Martinez et al.^20^ employed an LRP rule to highlight AF features. To explain classifications of AF, normal sinus rhythm (NSR), and left branch bundle blocks, Bender et al.^21^ employed multiple LRP rules and integrated gradients (IG). In contrast to that, Singh & Sharma^22^ conducted a more systematic comparison of four xAI methods based on Shapley additive explanations (SHAP), local interpretable model-agnostic explanations (LIME), and GradCAM. SHAP was also used in^23–26^ and exemplary compared to LIME and permutation feature relevance in^12^. Besides these *post-hoc* methods, applied to a model after classification, multiple works visualized *ante-hoc* generated attention layer values to explain ECG classifications, showing the samples’ relevance for classification^27–31^.

For validation of trustworthiness, most studies^4–6,18^ only qualitatively compare relevant regions according to xAI with diagnostic criteria and lack in quantitative validation. Studies focusing on the general explainability of time series classifiers by Schlegel et al.^32^ and Mercier et al.^33^ have examined the relevance of the machine-highlighted regions of time series data only for the machine itself. The method used for this purpose is called pixel-flipping, also known as perturbation or occlusion analysis^10^. By pixel-flipping the sample values are changed based on the relevance ranking of an xAI method. Perturbations following more truthful rankings will lead to faster decreases in a classification metric, identifying the best xAI methods. Previous results of this method showed no general preferable xAI method for different models or even for different datasets analyzed by the same model architecture^32,33^. Thus, for every new classification problem solved by a DL model, a validation of the sample relevance by different xAI methods needs to be conducted.

Besides the lack of interpretability, questionable reliability of classification performances also hinders the implementation into clinical practice. Many approaches (see Table 1) were trained solely on MIT-BIH datasets with small numbers of patients. Some limited the data and classification task to AF vs. NSR. Both points contribute to the concern of models not learning about the versatility of possible disease manifestations in everyday patients. But for clinical impact, a reliable DL ECG classifier must achieve high classification accuracy for unseen versatile test sets consisting of recordings from different individuals, that reflect the data variability in everyday patients. In addition to robust classification results, interpretable and trustworthy explanations for these classifications should exist to avoid misdiagnosis based on blind trust or to mitigate a general lack of trust in the classification results of the machine.

**Figure 1:**
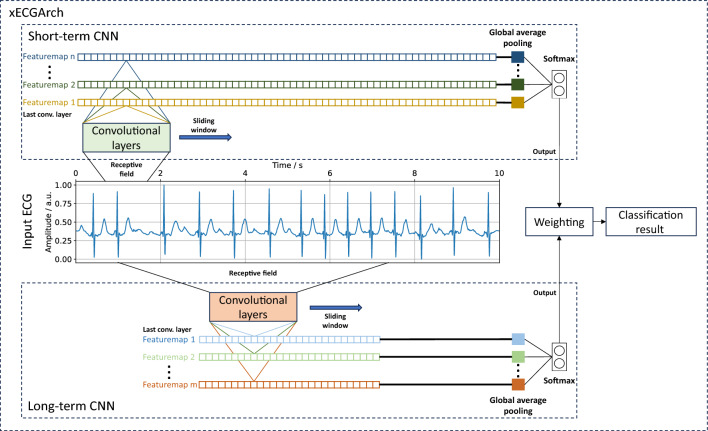
Visualization of the functionality of xECGArch. The short- and long-term CNN individually analyze and classify the ECG signal. Then a weighted average of both model outputs is calculated and the final classification result is determined by the combined softmax output with the highest value.

To overcome the limitations of previous approaches and to deliver a reliable, trustworthy, and interpretable DL architecture for ECG analyses, we present a novel architecture, the so-called xECGArch. We parameterized xECGArch for the application of AF detection in this paper. To achieve transferability from clinical application to home setting and thus reach a larger target group, we focused on single-lead ECGs^34,35^. Within xECGArch, we leverage global average pooling layers for signal analysis by independent CNNs on different time scales. Figure 1 depicts the general concept of xECGArch. The short-term CNN is designed to focus on morphological features at the beat level, while the long-term CNN is given the possibility to learn and recognize rhythmic patterns across multiple beats. This is implemented by receptive fields of different sizes. Both classifications are then analyzed independently by xAI methods and a final classification result is created by their weighted averaging. Our main contributions were as follows: (1) For the first time, a distinction of short- and long-term ECG analysis within a DL architecture, leading to a higher degree of result interpretability is possible; (2) The achievement of model reliability by optimizing the parallel structure of short- and long-term classification to detect AF in four publicly available datasets with various cardiovascular diseases and to test their performance on a test dataset with previously unknown recordings. We combined the CNNs into an ensemble within xECGArch to increase the detection performance; (3) First in ECG analysis, a comparison of 13 xAI methods for their trustworthiness by a novel perturbation scheme for pixel-flipping in addition to the prevalent perturbation scheme was conducted. The resulting most suitable xAI method was not deemed useful before in the ECG context and contrasts the works of others. Explanations generated by this method were coincidental with ECG features from textbook knowledge regarding AF detection. In line with expectations, the explanations of the short-term model seemingly emphasized morphological features and those of the long-term model rhythmic features.

## Results

### xECGArch parameterization and performance

**Figure 2:**
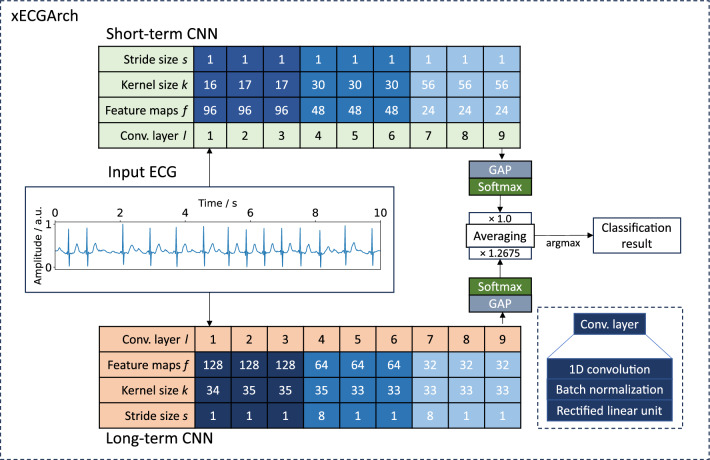
Proposed xECGArch parameterization for AF detection. Parameters *k* and *s* for the short- and long-term CNN show the solutions of the receptive field formula. Parameter *f* was found by hyperparameter optimization. The weighting factor was calculated by finding the optimal weight across all cross-validation folds. GAP, global average pooling; ConvLayer, convolutional layer.

We classified 10-s AF and non-AF ECGs obtained from four public databases. For this purpose, we implemented xECGArch consisting of a short- and a long-term CNN. Both CNNs share the same architecture with nine convolutional layers, including batch normalization and rectified linear unit activation (ReLu), followed by a global average pooling for feature calculation and a softmax layer of size two for classification. The individual designs were derived by solving Equation 1 of the receptive field of the final convolution layers concerning the input layer^36^. Currently no methodological approach is available for the choice of the CNN’s kernel size *k* and stride size *s* for the different convolutional layers *l*. Thus, we used an empirical solution for an exemplary parameterization, which is shown in Fig. 2. We parameterized the receptive field size $r_{short{\text {-}}term} = 300$ samples (0.6 s) and $r_{long{\text {-}}term} = 5000$ samples (10 s) to consider short- and long-term features. For the short-term model an interval of 0.6 s was chosen, to include maximum one heart beat at a frequency of 100 beats per minute, which is the upper boundary for healthy individuals at rest. In contrast a 10 s interval was chosen for the long-term model to cover beat changes over the whole signal.

**Table 2:** Cross-validation and test set metrics for the short- and the long-term model and xECGArch. Best scores achieved for each model during cross-validation are highlighted in bold.

Dataset	Model	Fold	Sensitivity	Specificity	Accuracy	F1-Score
Validation	Short-term	**1**	**94.90**	**93.70**	**94.30**	**94.32**
		2	93.55	93.14	93.34	93.34
		3	94.79	92.13	93.46	93.52
		4	93.55	94.03	93.79	93.76
		5	92.30	94.26	93.28	93.20
	Long-term	1	94.79	95.05	94.92	94.90
		2	96.71	93.25	94.98	95.05
		3	94.23	95.39	94.81	94.76
		**4**	**94.79**	**95.50**	**95.14**	**95.11**
		5	93.89	92.28	95.08	95.02
Test	Short-term	1	94.28	93.73	94.01	94.18
	Long-term	4	94.47	95.61	95.00	95.13
	xECGArch	–	94.87	95.82	95.33	95.43

Scores of the best hyperparameterized short- and long-term models found by the five-fold cross-validation are summarized in Table 2. For the short-term CNN, the best hyperparameters were a batch size of 32, a learning rate of 0.001, and 24 feature maps in the last convolutional layer. The long-term CNN classified best with a batch size of 8, a learning rate of 0.0001, and 32 feature maps in the last convolutional layer. On the unseen test dataset ($n=986$ ECGs), the best of the five short-term models reached a binary F1 score of 94.18%, an accuracy of 94.01%, a sensitivity of 94.28%, and a specificity of 93.73%. The best long-term model reached a binary F1 score of 95.13%, an accuracy of 95.00%, a sensitivity of 94.47%, and a specificity of 95.61%. By averaging the softmax output for each class from both models and taking the highest averaging class value as the classification result, both models were combined. For further optimization, the short- and the long-term model for every cross-validation fold were taken together and the optimal weighting of the model outputs for reaching the highest possible F1 Score was determined. By averaging the weight over all five folds, we defined the weight for the short-term model outputs in the combined model to be 1.0 and the weight for the long-term model to be 1.2675. This weight increased the binary F1 score to 95.43%, the accuracy to 95.33%, the sensitivity to 94.87%, and the specificity to 95.82%.

### Explanation validation

**Table 3:** Relative area under the curve (AUC) values for the different perturbation schemes and models. Bold cells mark the highest score per row and italic cells the second-highest. Methods that have not reached first or second rank once were omitted for readability. LRP, layerwise relevance propagation; DTD, deep Taylor decomposition; ITG, input times gradient.

Scheme	Model	LRP-$\alpha \beta$	LRP-Z	LRP-$\epsilon$	DTD	GradCAM+	ITG	Random
Interpolation	Short-term	0.248	0.217	0.220	**0.546**	*0.427*	0.270	0.129
	Long-term	**0.852**	0.230	0.168	*0.817*	0.601	0.240	0.087
Zero	Short-term	0.261	*0.490*	0.459	0.209	0.232	**0.529**	0.252
	Long-term	0.350	0.603	*0.660*	0.306	0.297	**0.712**	0.303

**Figure 3:**
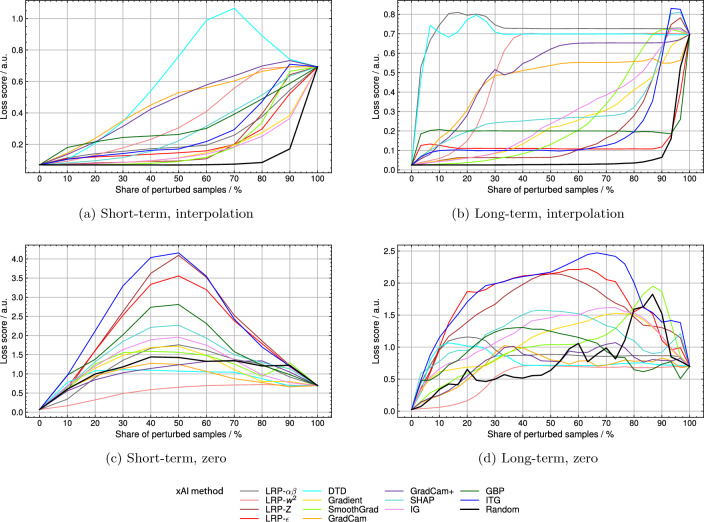
Pixel-flipping validation on 13 different xAI methods by the interpolation perturbation scheme for (**a**) the short-term model and (**b**) the long-term model and by setting zero perturbation scheme for (**c**) the short-term **d**) and long-term model. LRP, layerwise relevance propagation; DTD, deep Taylor decomposition; SHAP, Shapley additive explanations; IG, integrated gradients; GBP, guided backpropagation; ITG, input times gradient.

By pixel-flipping via linear interpolation deep Taylor decomposition (DTD) reached the highest relative area under the curve (AUC) of 0.546 for the short-term and the second-highest AUC of 0.816 for the long-term model (see Table 3). At the same time, LRP-$\alpha \beta$ reached an AUC of 0.247 and 0.852 for the short- and the long-term model, respectively. Loss score curves can be seen in Fig. 3a and b. The loss score of the short-term model shows a delayed reaction to the sample perturbation by DTD rankings. In the case of the long-term model, a steep increase in the loss score is reached by perturbation in order of DTD and LRP-$\alpha \beta$ rankings. For both models, the perturbation of randomly chosen samples did not change the loss score until around a perturbation percentage of around 70%. Relative AUC scores for random perturbation were 0.129 and 0.087 for the short- and the long-term model. When using the perturbation scheme of setting values to zero (see Fig. 3c and d), the order of decreasing relevance according to input times gradient (ITG) led to the biggest increase in loss score for both models, reaching relative AUCs of 0.529 and 0.712, respectively. With DTD relative AUCs of 0.125 and 0.306 were reached for the short- and the long-term model. The perturbation of randomly chosen samples changed the loss score for both models directly from the beginning and led to relative AUCs of 0.252 and 0.303, respectively.

### Qualitative and pseudo-quantitative explanations

**Figure 4:**
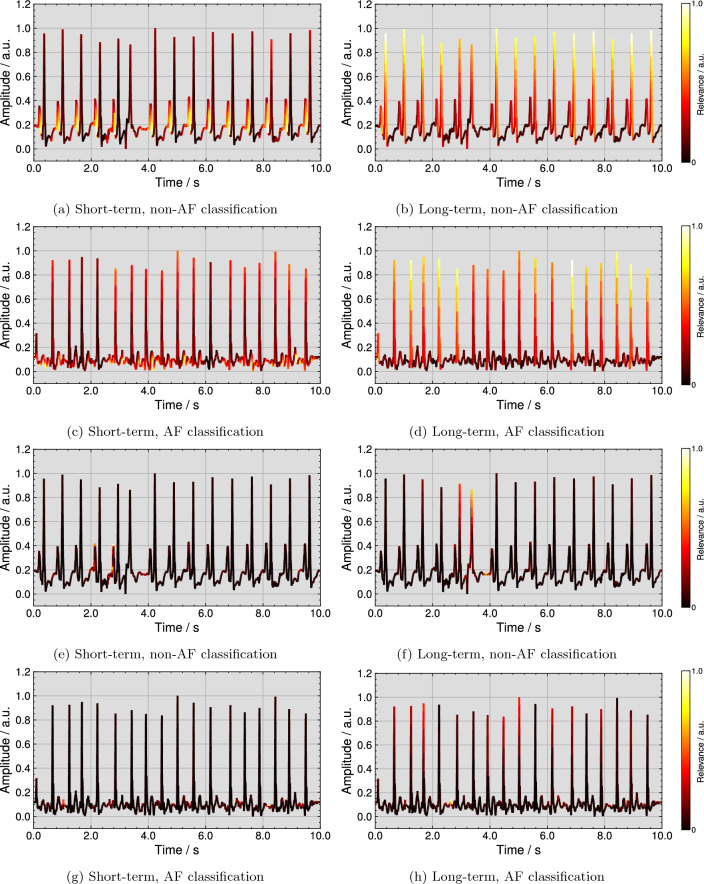
Deep Taylor decomposition (DTD) explanations for a correct non-AF classification by (**a**) the short-term and (**b**) the long-term model and for a correct AF classification by (**c**) the short-term and (**d**) the long-term model and input times gradient (ITG) explanations for a correct non-AF classification by (**e**) the short-term and (**f**) the long-term model and for a correct AF classification by (**g**) the short-term and (**h**) the long-term model.

Figure 4 (top row) visualizes examples for qualitative explanations by the DTD method for one correct classified non-AF and AF ECG each. Further examples from all four considered ECG databases for correct non-AF and AF classifications are publicly available^37^. As shown in Fig. 4a, the short-term model assigned the highest relevance scores to P wave flanks of all regular beats for the non-AF classification. The explanation in Fig. 4b highlights R peaks or regular rhythm as most important for the long-term model’s non-AF classification. In the case of AF, Fig. 4c shows that the short-term model pays the most attention to F waves. For the long-term model, R peaks are marked as most important in Fig. 4d, however, irregularly. Additionally, Fig. 4 (bottom row) visualizes examples for qualitative explanations of the same ECGs by the ITG method. In the non-AF example for the short-term model in Fig. 4e, two P waves of 14 regular beats are highlighted as most important and the area before the P wave onset is highlighted with the second-highest relevance for most beats. For the same ECG in Fig. 4f, the R peak of the extrasystole is marked as most important together with the pre-onset area of the following beat. For the AF classification, the explanations for the short- and the long-term model are shown in Fig. 4g and h. A small amount of F waves is highlighted as most important.

**Figure 5:**
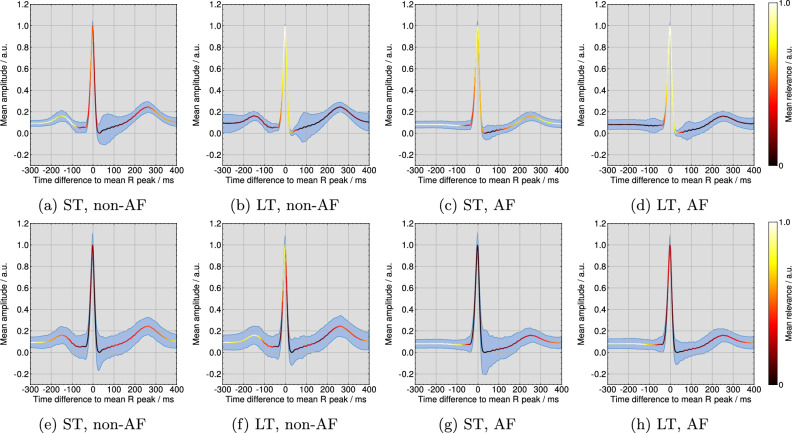
Pseudo-quantitative visualization of the mean relevance and the mean intra-ECG variation coefficient (shaded in blue) according to the deep Taylor decomposition (DTD) method for all (**a**) non-AF beats by the short-term model, (**b**) non-AF beats by the long-term model, (**c**) AF beats by the short-term model, and (**d**) AF beats by the long-term model and for the input times gradient (ITG) method for all (**e**) non-AF beats by the short-term model, (**f**) non-AF beats by the long-term model, (**g**) AF beats by the short-term model, and (**h**) AF beats by the long-term model.

In Fig. 5 (top row) the template beats over all ECGs are plotted with the pseudo-quantitative mean relevance and the mean intra-ECG variation coefficient per model for the DTD method. According to Fig. 5a, the short-term model focuses mainly on the P wave for non-AF classifications, while Fig. 5e demonstrates a high emphasis on the interval before the QRS complex and after the T wave and a secondary focus on the R peak for the AF classifications. Following Fig. 5b and d, the long-term model targets QRS complexes. Figure 5 (bottom row) illustrates the pseudo-quantitative mean relevance and the mean intra-ECG variation coefficient per model for the ITG method. For the short-term model in Figure 5e, the area of highest ITG relevance for non-AF classifications is found before the P wave and after the T wave. Figure 5g shows a focus on the short-term model in the pre-QRS complex interval. According to ITG in Fig. 5h and f, the long-term model targets on the pre-QRS complex area as most important, while QRS complexes themself are of secondary relevance. The mean intra-ECG variation coefficient of relevance was lower across DTD explanations than across ITG explanations, especially in areas of highest relevance.

## Discussion

The increase in classification performance through combined short- and long-term model outputs reveals that both models use different information for decision-making. Due to the high F1 Score of 95.43%, the combined model can be described as a reliable AF detector (see “xECGArch parameterization and performance” section). Compared to the classification metrics of the approaches reported in the literature (see Table 1), those of the model described are somewhat lower. But as described earlier, apart from the works of Cai et al.^38^ and Jo et al.^15^, previous publications used small datasets or distinguished only between AF and NSR, making the scores incomparable for generalization performance.

The validation of xAI methods by linear interpolation clarified that methods chosen in previous works (e.g. IG^21^, GradCAM^15^, LRP-$\epsilon$^20^) were unsuitable for explaining our models. Instead, explanations by DTD have been shown to be the most trusted for the short- and the long-term model on average, as they led to the highest and second-highest relative AUCs (see “Explanation validation” section). When perturbing sample values with the value zero, ITG led to the largest increases in loss score for both models while DTD led to much smaller scores. By this perturbation scheme, DTD would be deemed unsuitable, contrasting the results of the linear interpolation scheme. By interpolating random samples, the change in model performance was near to none for perturbing 70% of samples, while perturbing random sample values with zero led to an early and large increase in the loss score. Simultaneously relative AUCs were much bigger when setting random samples to zero. These results are in line with the concern of Hooker et al.^39^ that unsuitable perturbation schemes introduce noise that distorts the classification performance and makes it hard to attribute loss score changes to sample relevance or noise. Since the proposed method of interpolation does not influence the classification primarily when perturbing random samples, it might be superior for trustworthy xAI explanation validation. Thus, we choose DTD to explain our models. The perturbation scheme of interpolation is limited in that it reduces information to a baseline, which most likely represents one single class. For example, in the case of the short-term model, it is unlikely that perturbation of AF ECGs will lead to classification as non-AF because at no point in time P waves are inserted. An ideal perturbation scheme would extract the class characteristic features highlighted by the xAI method and exchange these features between classes to measure their influence (e.g. exchanging P waves with F waves and vice versa).

The DTD explanations are well interpretable in that they highlight known clinical markers for the distinguishment of non-AF from AF. In the case of the short-term model, the exemplary explanation highlights the existing P waves in non-AF ECGs, while mainly F waves are highlighted in the AF ECG (see “Qualitative and pseudo-quantitative explanations” section). This behavior is further emphasized by the mean relevance score per beat projected on class representing beats and the low mean intra-ECG variation coefficient in areas of high relevance. While in the non-AF beat the focus lies on the P wave, the relevance scores are wider distributed across the AF beat, reflecting the possible beat-wide distribution of F waves. The explanations for the long-term model showed a relatively even distribution of relevance among even-spaced QRS complexes in non-AF ECGs, while anomalous beats that did not fit the local rhythm were allocated a lower relevance. In AF ECGs, the long-term model showed the inverse behavior and irregularly timed QRS complexes were deemed most important. This and the visible focus on the QRS complex in the class representative beats point to the long-term model using the heart rhythm as the main feature for classification. The ITG explanations showed similar behavior in that no QRS complexes were highlighted as important for the short-term model classifications, but some for the long-term model classification (see Fig. 5). However, these explanations did not appear consistent in the highlighted features. Only some P or F waves were of higher relevance. This effect appears to be confirmed by the higher mean intra-ECG variation coefficient across relevant areas. Marked QRS complexes did not appear to represent rhythmic features for the long-term model. The ITG mean relevance per class representative beat did not indicate the preferred use of P waves by the short-term model or QRS complexes as the most relevant feature for the long-term model for classification.

Another indication that the models use the described features highlighted in DTD explanations is the course of the loss score during explanation validation by linear interpolation. For the short-term model, the maximum loss score is reached after perturbing 70% of the samples. A possible explanation is, that this model uses features that are spread more widely over the signal than others, for example, F waves. In this case, F waves are still found in the signal, even if many samples have been smoothed by interpolation, making AF detection still possible. In contrast, the long-term model’s steep increase in loss score indicates that the model uses less frequently occurring features. It is conceivable, that the removal of QRS complexes distorts the rhythm and thus influences the classification. However, further statistical evaluation is needed.

Besides ECG analysis, xECGArch is applicable to other quasi-periodic biosignals or medical time series analysis due to the generalized architectural design. Short- and long-term features are dominant in most cardiovascular biosignals like the photoplethysmogram or continuous blood pressure. However, the CNNs of xECGArch need a reparameterization of the receptive field sizes and hyperparameters to achieve trustworthy results. Together with the process of trustworthy xAI validation, xECGArch is also usable to investigate manifestations of arbitrary classes in medical time series. A possible scenario is the classification and differentiation of a healthy collective from a collective suffering from a poorly researched disease, to find the deviating signal regions in the diseased collective. It is also conceivable to use xECGArch as a starting point for investigations to better understand subgroups in physiological recordings. The architecture could, for example, learn to differentiate patient characteristics (e.g. sex or age) in medical time series and thus, the explanations could deliver clues for a better group-adapted medicine.

xECGArch classification explanations present, to the best of our knowledge, the first trustworthy ECG explanations that allow by architectural design the differentiation of short- and long-term features. ECG explanations can be validated qualitatively and pseudo-quantitatively, separated to short- and long-term features, to uncover indications of so far unknown pathophysiologcial ECG patterns. This might bring clinicians to bridge the gap between AI explanations by xECGArch and existing pathophysiological knowledge to explain the behavior of unsolved diagnostic problems. However, pseudo-quantitative validation need to extended by projections of ECG delineations to enforce a more accurate validation over multiple variations in ECG morphology. Nonetheless, xECGArch allows clinicians to interpret trustworthy explanations by guiding the eyes to understand their classification. Thus, they can be used to speed up ECG screening of longer recordings or for consultation in differential diagnosis. A future diagnosis support system could also benefit from a combination with concept-based approaches. Concept-based classifiers are trained to initially identify individual characteristics, such as the morphology of P waves or rhythmicity, utilizing extensively annotated data. Subsequently, they establish connections between these features for a disease diagnosis^15,40^. xECGArch allows the differentiation of rhythmic and morphological features by design and thus opens a new level of analysis based on the clinical reading of biosignals. This could improve the detection of morphological or rhythmic concepts, as well as the explainability of the detection and therefore the trustworthiness of disease diagnosis.

Besides in-hospital diagnostics, out-of-hospital surveillance of the patients cardiovascular system is of high interest. The use of ambulatory ECG systems and smartwatches creates large amounts of data, offering high potential for disease detection in everyday situations^34^. However, one of the main challenges for population-wide cardiac screening is the availability of exhaustive expertise^41^. Implementations of trustworthy automated disease detection algorithms, like xECGArch, might be a major contributor in the analysis of cardiac biosignals in big data.

In further research, we will focus on the application of xECGArch to other medical time series and extended quantitative validation for the application to unsolved diagnostic problems in cardiovascular medicine.

## Methods

### Data material

Four 12-lead ECG databases with a sampling frequency of 500 Hz were included: PTB-XL^42,43^, Georgia-12-Lead^44^, China Physiological Signal Challenge 2018 (CPSC2018)^13^ and Chapman-Shaoxing^45,46^. We solely utilized lead II as it is generally applicable for mobile measurement devices and suited for AF detection^47,48^. Because the databases PTB-XL, Chapman-Shaoxing, and Georgia-12-Lead only contain 10-s recordings, while the CPSC2018 database’s median recording length was 12 s, we only used ECGs with at least 10 s of recording time. From recordings longer than 10 s, the 10-s window segment from the middle of the ECG was used. In sum, 4927 AF-recordings and 43,574 non-AF-recordings from all datasets were available. Because training on unbalanced datasets can lead to classifiers that unreliably predict the underrepresented classes, our datapool was limited to 4927 AF and 4927 randomly selected non-AF recordings.

**Table 4:** Number of ECGs for the different classes over the four datasets in the used datapool. NSR, normal sinus rhythm; ST, sinus tachycardia without other annotations; SB, sinus bradycardia without other annotations.

Class	Database				
	PTB-XL	Georgia	CPSC2018	Chapman-Shaoxing	Total
NSR, ST, SB	290	67	12	123	492
OTHER	169	2032	1921	313	4435
AF	1497	553	1097	1780	4927

Moreover, the amount of normal sinus rhythm, sinus tachycardia, and sinus bradycardia without further disease label instances was balanced to 492 of the 4927 non-AF recordings to reduce the effects of overrepresentation in the datapool. The distribution over datasets can be found in Table 4. Of all recordings, 90% were used for training (4448 non-AF, 4420 AF) and 10% for testing (479 non-AF, 507 AF). The training data was randomly split into five folds for cross-validation.

### Data preprocessing

ECG signals were high-pass filtered with a 4th-order Butterworth filter with a cut-off frequency of 0.3 Hz, realized as a 2nd-order section filter cascade^49^. For noise reduction, a discrete wavelet transformation (DWT) approach was applied with eight levels and the sym5 wavelet^50^. To remove edge effects, we multiplied ECG signals with a Tukey window with alpha = 0.06. Subsequently, signals were scaled between 0 and 1 for faster network convergence.

### Network architecture

ECG interpretation consists of multiple strategies, e.g., analysis of morphology and heart rhythm. On this basis, we propose a novel trustworthy, interpretable deep learning architecture, that contains two independent networks that are capable of carrying out a short-term or a long-term analysis. This is achieved by designing 1D CNNs with varying receptive field sizes of the neurons in the last convolutional layers regarding the input signal. They are realized by defining for different layers *l*, stride sizes *s*, and kernel sizes *k* to solve for a receptive field size *r* in the formula ^36^

1 $$\begin{aligned} {\displaystyle r = \sum _{l=1}^{L} \biggl ( (k_{l} - 1) \prod _{i=1}^{l-1} s_{i} \biggr ) + 1 }. \end{aligned}$$

The short-term network was limited to a receptive field in the last convolutional layer of $r_{short-term} = 300$ samples regarding the input signal. Hence, a neuron in the last convolutional layer can only connect temporal information in a time window of 0.6 s. In contrast, the long-term network with a receptive field of $r_{long-term} = 5000$ samples or 10 s can connect information over the whole 10 s of an ECG recording.

In both networks, the feature maps of the activation after the last convolutional layer are averaged by global average pooling (GAP). GAP outputs are used as input to a softmax layer for classification. GAP is essential for allowing networks with smaller receptive fields on larger input data because it allows a dimension reduction of the input to the following classification network. The proposed xECGArch consists of a single softmax layer. Commonly in CNN without GAP the feature maps of the last convolutional layers are flattened into a single vector of a dimensionality indirectly proportional to the receptive field of the last convolutional layer. This results in an increased number of learnable weights, which increases the learning time and possibly degrades performance. To further reduce the dimensionality of the input to the softmax layer, the number of feature maps was reduced with the depth of the network. The first through third layers were set to have four times as many feature maps as the seventh through ninth layers, and the fourth through sixth layers were set to have twice as many. Before inserting the ECG signals into the network, model-specific padding was applied to ensure that all data points at the edges were sampled as often as the data points in the center of the signals. The beginning of the signal was padded with a vector of repetitions of the first signal value and the end with a vector of repetitions of the last signal value. The size of the padding vector was congruent with the size of the receptive field *r* of the last convolutional layers of the network regarding the input signal.

Each CNN was subjected to a five-fold cross-validation grid search to find the most appropriate hyperparameters. Explored hyperparameters included the batch size = [4, 8, 16, 32], learning rate = [0.001, 0.0001, 0.00001], and the number of last layer feature maps = [8, 16, 24, 32]. All models were optimized by using the Adam optimizer with categorial cross-entropy being used as a loss function^51^. The implementation was realized in Keras for TensorFlow 2.10^52^.

The mean and standard deviation of the F1 score over all five folds were calculated for each hyperparameter combination. Then, the best hyperparameters were selected by searching for the combination that achieved the highest score for the metric $F1_{mean}-F1_{std}$ over the five folds. By subtracting the standard deviation from the mean, hyperparameter combinations were eliminated if fold values varied over a wide range, which makes them difficult to consider reliable.

### Network interpretation

Multiple model-specific explanation methods were used: gradient-based methods vanilla gradient^9^ with absolute value postprocessing, IG^53^ with 32 integration steps and absolute value postprocessing, ITG^54^, guided backpropagation^55^, SmoothGrad^56^ with a noise scale of 10%, 32 heatmap augmentations and absolute value postprocessing, and the attribution-based methods DTD^57^ and LRP^10^ in the form of LRP-$\epsilon$  with $\epsilon = 0.1$, LRP-Z, LRP-$\alpha \beta$ with $\epsilon = 0.1$ and LRP-$w^2$. Explanation methods were implemented in the iNNvestigate 2.0 Toolbox^58^. Additionally, the model-specific methods GradCAM^59^ and GradCAM+^60^, which use gradient and activation information, were applied. In addition, the model agnostic method SHAP was used in the form of a gradient explainer, where 500 recordings were used as a background dataset for value exchange^61^.

Gradient-based methods can be understood as a sensitivity analysis of the network output *f*(*X*) for the input components $X = [x_{1},x_{2}, ... ,x_{n}]$, where the gradient

2 $$\begin{aligned} \nabla f(X) = \sum _{i=1}^{n}\Biggl |\frac{\partial f(X)}{\partial x_{i}}\Biggl |\times \textbf{e}_{i} \end{aligned}$$

describes how sensitive the classification function *f*(*X*) is to changes of a component $x_{i}$. $\textbf{e}_{i}$ represents the unit vector that spans a coordinate space. Decomposition methods like LRP and DTD seek to redistribute the network output to the input components by so-called relevance propagation rules (RPR). LRP and DTD are deeply intertwined. DTD is a mathematical framework for the generation of meaningful RPRs. To distribute the relevance to different neurons from layer to layer the following formula is used:

3 $$\begin{aligned} R_{i} = \sum _{j} \frac{\partial R_{j}}{\partial x_{i}}|_{\tilde{x_{i}}^{(j)}}(x_{i}-\tilde{x_{i}}^{(j)}) \ . \end{aligned}$$

Hereby the relevances $R_j$ of all neurons (or neuron outputs) $x_j$ in a layer are summed up to calculate the relevance $R_i$ of one neuron $x_i$ in the previous layer. This is done for ever neuron in every layer, including the input layer. Specific RPRs are realized by the choice of the root point $\tilde{x_{i}}^{(j)}$, which should be picked based on the input structure of the Network Layer^62^. Multiple, but not all RPRs of LRP can be derived in the DTD framework. DTD explanation differ from all other explanations, as they only evaluate to which degree features speak positively for a classification and not how they oppose it. In GradCAM, a convolutional layer, in most cases the last one, is first selected. Then, a weight $w_k$ is defined for every feature map $A_k$ of the layer by averaging over all the gradient values of the feature map of length *Z* regarding the classification y:

4 $$\begin{aligned} w_{k} = \frac{1}{Z} \sum _i \frac{\delta y}{\delta A_{k}^{i}} \ . \end{aligned}$$

The positive contribution of all feature maps is then summed up into the heatmap

5 $$\begin{aligned} L = ReLU \left( \sum _{k} w_{k} A_k \right) \ . \end{aligned}$$

The heatmap can then be upsampled and projected onto the original input data. GradCAM+ differs from GradCAM such, that only positive gradients contribute to the weight:

6 $$\begin{aligned} w_k = \frac{1}{Z} \sum _i ReLU\left( \frac{\delta y}{\delta A_{k}^{i}}\right) \ . \end{aligned}$$

SHAP is an approximative implementation of Shapley values^63^ from game theory. Shapley values denote the contribution $\phi _i(v)$ of every player *i* to a game outcome, by evaluating all possible coalitions S of m players, that do not contain player *i*:

7 $$\begin{aligned} \phi _i(v)=\frac{1}{m} \sum _{S \subseteq M \backslash \{i\}}\left( \begin{array}{c} m-1 \\ |S| \end{array}\right) ^{-1}(v(S \cup \{i\})-v(S)) \ , \end{aligned}$$

where the term $v(S \cup \{i\})-v(S)$ containing the characteristic function *v*(*S*) denotes how much the coalition gains by cooperating with player *i*. This mindset can be adapted to features and classification results. But features can not be simply omitted to calculate incomplete coalitions, because classifiers often rely on complete feature vectors. Instead, with SHAP, the values of an absent to a coalition-denoted feature get replaced with different values from the underlying dataset, and the difference between the original classification and the classification on the coalition without the original feature value is measured over multiple value exchanges.

### Explanation validation

Different methods of interpretation may provide different explanations. To analyze which method can generate the most truthful explanations, pixel-flipping was applied to the sample ranking^10^. By pixel-flipping, samples are perturbed according to the ranking of the interpretation method. Perturbing the order to a truer ranking results in a steeper drop in classification performance or an increase in loss points when reclassifying the perturbed signals. In this work, a new perturbation scheme has been developed to address the concern that pixel-flipping degrades classification performance because the perturbation injects noise that is unknown from the training data distribution^39^. Thus, sample values according to the rankings were extracted and the missing values were linearly interpolated. With this concept, the lower frequency data structure is preserved, while higher frequency information is destroyed without adding obvious noise. To estimate the influence of noise introduced by different perturbation schemes, classification performance after perturbation of randomly chosen samples has been evaluated. When a high classification score or low loss score can be achieved after perturbing a large number of samples, it can be concluded that the perturbation schemes introduce little noise. To show the advantage of the new perturbation scheme, a comparison to the frequently used scheme of setting values zero was applied. The latter, of course, introduces noise because the models were not trained on data with sudden jumps to zero. For quantification purposes, the AUC concerning the area spanned by the maximum loss score and the share of perturbed samples was calculated.

For qualitative validation of explanations, relevance values of the best method per perturbation scheme were projected onto exemplary ECGs. Furthermore, template beats were created for every recording by two-dimensional signal warping^49^. Similarly, the mean relevance and standard deviation of relevance per template beat per recording were calculated. Template beats, mean relevance, and standard deviation of relevance were separately averaged across all recordings of the non-AF and AF classes. Mean relevance was directly projected onto the mean template beats for pseudo-quantitative validation. The intra-ECG variation coefficient of relevance, describing the ratio of standard deviation to the mean, was illustrated as a range around the class representative template beat. For better pseudo-quantitative visualization, the intra-ECG variation coefficient of relevance was scaled down to one-tenth its size.

## Data Availability

The results presented in this study are based on data from publicly available databases. Explanations of xECGArch classifications from all four considered ECG databases for correct non-AF and AF classifications are publicly available under CC BY 4.0 license^37^. Additional data supporting the findings of this study are available from the corresponding author upon reasonable request.

## Code availability

Due to the patent application DE 10 2023 118 246.3 (submitted), which covers parts of the methods presented in this paper, the code cannot currently be made publicly accessible.
